# *bla*_OXA−51_-Negative *Acinetobacter calcoaceticus–baumannii* Complex as a Cause of Human Infection in Peru

**DOI:** 10.1155/jotm/8851906

**Published:** 2025-11-29

**Authors:** Rosario Oporto-Llerena, Rosario Huerto-Huánuco, Yaneth Quispe-Hualpa, Luciano A. Palomino-Kobayashi, Gabriela Soza, Jesús Rojas-Jaimes, Patricia Gonzales, Luis Pollack, Andrea C. Gomez, Gina Salvador-Luján, Edwin Cuaresma, Nestor Luque, Martín Casapia, Kovy Arteaga-Livias, Yolanda Sáenz, Maria J. Pons, Joaquim Ruiz

**Affiliations:** ^1^Research Group on Dynamics and Epidemiology of Antimicrobial Resistance—“One Health”, Universidad Cientifica del Sur, Lima, Peru; ^2^Microbiology Service, Instituto Nacional Materno Perinatal, Lima, Peru; ^3^Faculty of Health Sciences, Universidad Privada del Norte, Lima, Peru; ^4^Human Health School, Faculty of Health Sciences, Universidad Cientifica del Sur, Lima, Peru; ^5^Service of Infectious Diseases, Hospital Maria Auxiliadora, Lima, Peru; ^6^Laboratory of Microbiology, Hospital Nacional PNP Luis N. Sáenz, Lima, Peru; ^7^Center for Basic and Translational Research AUNA Ideas, Lima, Peru; ^8^Laboratory of Microbiology, Hospital Militar Central, Lima, Peru; ^9^Laboratory of Microbial Ecology, Universidad Nacional Mayor de San Marcos, Lima, Peru; ^10^Laboratory of Clinical Pathology, Hospital III Daniel Alcides Carrión, Tacna, Peru; ^11^Human Health School, Faculty of Health Sciences, Universidad Peruana Union (UPeU), Lima, Peru; ^12^Intensive Care Unit, Hospital Emergencias Ate Vitarte, Lima, Peru; ^13^Service of Infectious Diseases and Tropical Medicine, Hospital Regional de Loreto, Iquitos, Peru; ^14^Faculty of Human Medicine, Universidad Nacional de la Amazonia Peruana, Iquitos, Peru; ^15^Facuty of Health Sciences, Universidad de Huánuco, Huánuco, Peru; ^16^Molecular Microbiology Area, Center for Biomedical Research of La Rioja, Logroño, Spain

**Keywords:** *Acinetobacter* spp., antibiotic resistance, community-acquired infection, hospital-acquired infection, OXA-51

## Abstract

**Background:**

Common identification techniques do not differentiate among members of the *Acinetobacter calcoaceticus–baumannii* (ACB) complex, and the presence of non-*baumannii Acinetobacter* is often misinterpreted. The *bla*_OXA−51_ gene is located within the chromosome of *Acinetobacter baumannii*. Despite its plasmid dissemination to other members of the genus, it may be considered in initial species screening. Thus, this study aimed to determine the presence of *bla*_OXA−51_-negative *Acinetobacter* spp. as a cause of infection in Peru.

**Methods:**

Two hundred ninety-eight ACB complex isolates from different regions of Peru were isolated between January 2018 and March 2024. Of these, 272 and 25 were confirmed as hospital-acquired and community infections, respectively. The presence of *bla*_OXA−51_ was determined by polymerase chain reaction, and the susceptibility levels to 12 antimicrobial agents were determined.

**Results:**

The results showed that 38 (12.7%) isolates were *bla*_OXA−51_-negative. These isolates were frequent among community infections (13/25, *p* < 0.0001), often causing urine infections. They showed significantly lower levels of resistance to almost all antimicrobial agents tested, and most of them were recovered from regions outside metropolitan Lima.

**Conclusion:**

A relevant number of infections by non-*baumannii Acinetobacter* species in Peru is suggested, highlighting the need for systematic identification of these species in the country.

## 1. Introduction

*Acinetobacter* spp. is a ubiquitous genus, which includes pathogenic microorganisms often isolated as a cause of severe infections, with *Acinetobacter baumannii* being the most relevant worldwide [1, 2]. *A. baumannii* has been included among the so-called *Enterococcus faecium*, *Staphylococcus aureus*, *Klebsiella pneumoniae*, *A*. *baumannii*, *Pseudomonas aeruginosa*, and *Enterobacter* spp. (ESKAPE) microorganisms because of its relevance as a cause of human infections as well as the high levels of antimicrobial resistance often presented by this microorganism [3], challenging current therapeutic schemes [4]. In this sense, carbapenem-resistant *A. baumannii* has been qualified as a pathogen of critical relevance by the World Health Organization (WHO) [4]. Nevertheless, it should be mentioned that a series of closely related species are biochemically indistinguishable from *A. baumannii*. Similarly, several automatized diagnostic tools, such as MicroScan WA or VITEK, among others, are also unable to differentiate these bacterial species [2, 5, 6]; this problem results in the use of the term *Acinetobacter calcoaceticus–baumannii* (ACB) complex to collectively refer to these indistinguishable species [2]. Different techniques have been evaluated to differentiate among the species of the ACB complex, including amplified ribosomal DNA restriction analysis (ARDRA) or, more recently, the use of matrix-assisted laser desorption/ionization time-of-flight (MALDI-TOF) [2, 7, 8]. However, these techniques are difficult to implement in routine bacterial identification, or are expensive and not available in different areas, especially in low- and middle-income countries, such as Peru.

The detection of specific chromosomal markers has been used in the identification of several microorganisms [9, 10]. In this regard, the detection of *bla*_OXA−51_-like genes, which are encoded in the chromosome of *A. baumannii*, has been proposed for preliminary differentiation of *A. baumannii* from related species [11]. However, it should be considered that the presence of these genes may not be fully accurate for the correct identification of *A. baumannii*, because a few members of the genus, such as *Acinetobacter pittii*, have acquired plasmid-borne *bla*_OXA−51_-like genes [12]. Meanwhile, their absence may be considered as a good marker to define a non-*baumannii Acinetobacter* spp. (NBA), although genetic events, such as *bla*_OXA−51_-like (hereafter referred to as *bla*_OXA−51_) gene disruptions related to the presence of insertions, may hinder the detection of this gene [13].

*A. baumannii* clinical isolates often exhibit high levels of antibiotic resistance, including last resort antibiotics [14]. This finding, together with the impact of *A. baumannii* infections on final patient outcomes, results in early antibiotic treatments, prior to knowing the antibiotic susceptibility data. On the other hand, the NBA often are susceptible to a variety of antimicrobial agents and usually exhibit lower virulence levels [15]. Thereby, an early screening test that is able to differentiate NBA from *A. baumannii* would result in better patient management as well as in a more rational use of antibiotics. In Peru, only sporadic reports of the presence of NBA have been described [7]. Thus, the present study aimed to determine the presence of non-*bla*_OXA−51_–producing *Acinetobacter* spp. as a cause of human infection in Peru.

## 2. Materials and Methods

**Figure 1:**
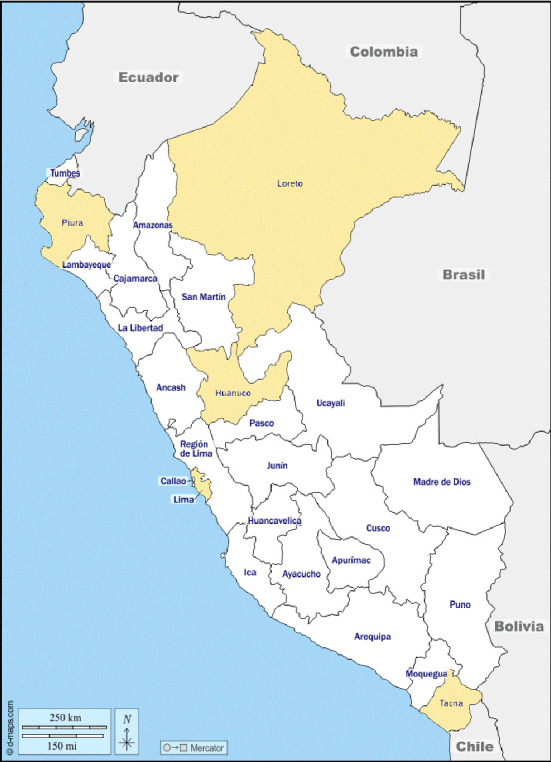
Map of Peru. In the map, the regions from which isolates were recovered are highlighted. Lima and Callao co-form the so-called “metropolitan Lima.” Iquitos is a city in the Loreto region. In Table 1, the number of isolates recovered in each region is indicated. Maps modified from https://d-maps.com/carte.php?num_car=4766%26lang=es (accessed on August 8, 2025).

Two hundred ninety-eight ACB complex isolates, from a variety of clinical sources, identified by automatized methods (VITEK-2, bioMérieux, Marcy l'Etoile, France) and/or biochemical determination were isolated between January 2018 and March 2024. The isolates were recovered in 14 Peruvian hospitals from metropolitan Lima, Northern, Southern, and Central Peru; two isolates from metropolitan Lima but with no exact origin were also included (Figure 1). All isolates were stored in skim milk (OXOID, Thermo Fisher Scientific, Waltham, USA) at −80°C until use. Hospital-acquired infections (HAIs) were defined as those manifested > 48 h after admission [14].

Isolates were grown on MacConkey agar (OXOID), and bacterial DNA was obtained by the boiling method, as described elsewhere [8], and 1.5 μL was used in each PCR reaction. In all cases, PCRs were prepared at a final volume of 25 µL (*bla*_OXA−51_) or 50 μL (16S rRNA and *rpoB*) containing dNTPs (0.2 mM each), primers (0.5 µM each), MgCl_2_ (2.5 mM), PCR buffer (1X), and TaqDNA polymerase (1U), and resolved in a 1.5% agarose gel stained with GreenSafe DNA Gel Stain 5% (Canvax, Cordoba, Spain). The presence of the *bla*_OXA−51_ genes was determined by amplifying a 353-bp fragment using previously described primers (5′-TAATGCTTTGATCGGCCTTG-3′ and 5′-TGGATTGCACTTCATCTTGG-3′) and conditions: 94°C × 5 min, 30 × (94°C × 25 s, 52°C × 40 s, 72°C × 50 s), and 72°C × 6 min [16]. The *A. baumannii* ATCC15308 was used as a positive control. Randomly selected amplified products were gel recovered using the E.Z.N.A Gel Extraction Kit (Omega Bio-Tek, Norcross, GA) and sequenced.

In a subset of 30 isolates (c. 10%), including 10 *bla*_OXA−51_-negative and 20 *bla*_OXA−51_-positive isolates, the bacterial identity was determined by MALDI-TOF (MALDI Biotyper, Bruker Daltonics GmbH & Co. KG, Bremen, Germany) and interpreted by the MBT Compass Library V11.0.0.0 (July 2021) [7], along with amplification and subsequent sequencing of 16S-rRNA and *rpoB* gene. Amplifications of 16s-rRNA and *rpoB* were performed using established primers 8F (5′-AGAGTTTGATCCTGGCTCAG-3′), 1510R (5′-GGTTACCTTTGTTACGACTT-3′), Ac696F (5′-TAYCGYAAAGAYTTGAAAGAAG-3′), and Ac1093R (5′-CMACACCYTTGTTMCCRTGA-3′), respectively, under previously described conditions [8, 17].

Antimicrobial susceptibility to ampicillin plus sulbactam, piperacillin plus tazobactam, cefotaxime, ceftazidime, cefepime, imipenem, meropenem, ciprofloxacin, levofloxacin, gentamicin, and amikacin was determined by disk diffusion, while colistin susceptibility was established by microdilution, in both cases in accordance with the Clinical Laboratory Standards Institute guidelines [18].

The isolates were classified by origin (either metropolitan Lima or other regions) and time of isolation as pre-COVID-19 pandemic (2018–2019), COVID-19 pandemic (2020–2021), and post-COVID-19 pandemic (2022–2024).

Intermediate and resistant isolates were categorized together as nonsusceptible for statistical purposes. Two *bla*_OXA−51_-positive isolates, for which no data about community or HAI origin were found, were excluded from the analysis related to this issue.

GraphPad software (https://www.graphpad.com/) was used for statistical analysis. The Fisher's exact test was used to establish the presence of significant differences (*p* < 0.05).

The study was approved by the Ethics Committee of the Universidad Científica del Sur (code: 059-2023-PRO99).

## 3. Results

Overall, 298 *Acinetobacter* spp. were recovered. Of these, 38 (13.0%) were negative for the presence of *bla*_OXA−51_ and were thereby likely NBA. These *bla*_OXA−51_-negative isolates were present in 11 out of 14 hospitals participating in the study, being recovered from all the Peruvian regions included in the study, but, although nonsignificant, they were most frequently from outside metropolitan Lima (31/267%–11.6% vs. 7/31%–22.6%; *p*=0.0912) (Table 1). The data showed that *bla*_OXA−51_-negative isolates were mostly from post-COVID-19 pandemic years (2022–2024), accounting for 32 out of 132 (24.2%) isolates recovered in this period (Tables 1 and 2).

When a subset of 29 isolates was fully identified, the results showed full concordance among 16S-rRNA, *rpoB*, and *bla*_OXA−51_ results, while MALDI-TOF results were discordant in 11 isolates (Table 3).

Two hundred seventy-one isolates were classified as HAIs, while 25 were from community origin. In two cases, no information was recovered. The most common samples were urine (14 cases, 56%) and respiratory (7 cases, 28%) among community isolates, and respiratory (127 cases, 51.2%) and blood (56 cases, 20.7%) among HAI (Table 4). Respiratory infections were most common among HAI (*p*=0.0224), while urine infections were most common among community samples (*p* < 0.0001) (Table 4).

The *bla*_OXA−51_-positive ACB complex accounted for 246 out of 271 (90.8%) isolates from HAIs, and for 12 out of 25 (48.0%) isolates from community-acquired infections, a difference statistically significant (*p* < 0.0001) (Table 4).

Concerning the antimicrobial susceptibility analysis, the *bla*_OXA−51_-negative isolates showed significantly lower levels of nonsusceptibility to all the tested antimicrobial agents, except for cefotaxime and colistin, than *bla*_OXA−51_-positive isolates (Table 5). Thus, among the *bla*_OXA−51_-negative isolates, no antimicrobial agent except cefotaxime (89.5% of nonsusceptibility) reached levels of nonsusceptibility greater than 50%, while, apart from colistin, ampicillin plus sulbactam was the most active among the *bla*_OXA−51_-positive isolates, with 58.1% of nonsusceptibility. Of note, the presence of a high number of ampicillin plus sulbactam (86 isolates), cefepime (60 isolates), and cefotaxime (26 isolates) intermediate isolates was detected among the *bla*_OXA−51_-positive group (Table 5). Fifty-three isolates showed resistance to colistin, 47 of them being *bla*_OXA−51_-positive (Table 5).

The origin (community vs. hospital) of the isolates was also reflected in differences in the resistance levels, with those related to HAIs exhibiting consistently significantly higher levels of resistance with *p* < 0.0001, excepting ampicillin plus sulbactam (*p*=0.0004) and colistin (*p*=0.363). Regarding colistin, all but one resistant isolate was recovered from HAIs, with the community isolate being a *bla*_OXA−51_-positive isolate recovered from a maternal milk sample. Thus, resistance levels to piperacillin plus tazobactam, imipenem, and meropenem were significantly higher among *bla*_OXA−51_-negative isolates, from HAIs. Meanwhile, *bla*_OXA−51_-positive isolates from HAIs were significantly more resistant to all tested antibacterial agents, but colistin, than their counterparts from the community (Table 6).

## 4. Discussion

The present study highlights the presence of a relevant number of infections associated with *bla*_OXA−51_-negative *Acinetobacter* spp. and confirms the presence of relevant differences among isolates possessing *bla*_OXA−51_ or not.

Infections due to NBA are less studied than those associated with *A. baumannii*, likely due to the inherent difficulties in differentiating the species of the ACB complex, which are usually misidentified, and reported as the ACB complex or often as *A. baumannii* [2]. Nevertheless, differences in both the prognosis and characteristics of infections by different members of the ACB complex have been described. Thus, the study by Wisplinghoff et al., which analyzed the impact of *A. baumannii*, *Acinetobacter nosocomialis*, and *A. pittii* causing bloodstream infections, reported that around one-third of infections were related to *A. nosocomialis* and *A. pittii*, with those related to *A. baumannii* presenting a bad prognosis [19]. Along this line, marked differences between bacteremic nosocomial pneumonia related to *A. baumannii* and *A. nosocomialis* infections have been described [20]. In addition to bad outcomes, including higher rates of leucopenia or leukocytosis, these differences include a higher frequency of pulmonary lobar consolidation, lower hemoglobin levels, and reduced platelet counts in patients with infections by *A. baumannii* [20]. These findings led to the suggestion that bacteremic nosocomial pneumonia by *A. baumannii* and *A. nosocomialis* are different clinical entities [20]. Of note, differences among infections by different NBA have also been described, with infections related to *A. pittii* requiring the need for fewer invasive procedures and intensive care unit admission than those related to *A. nosocomialis* [21]. All these findings indicate that early correct identification of an ACB complex member causing infection may contribute greatly to better patient management.

Current data show the presence of differences in the etiology of community and HAI *Acinetobacter* spp. infections, as well as in the type of infection related to these origins, with those *bla*_OXA−51_-negative being the most common among community infections, often as a cause of urine infections, and *bla*_OXA−51_-positive among HAI, mostly from respiratory or blood samples. These findings might be related to the virulence traits of *bla*_OXA−51_-negative and *bla*_OXA−51_-positive; in this line, it has been showed that species such as *A. pittii*, while possessing a series of virulence factors, often need additional factors to be horizontally acquired to become efficient nosocomial pathogens [15]. This, together with the present results, reflects the best adaptation of *A. baumannii* to nosocomial environments, but highlights the need to reinforce the correct identification of *Acinetobacter* spp. causing community infections. While most studies are focused on *A. baumannii* or do not differentiate amongst those causing infections [21–23], NBA have been described as a cause of community infections, but commonly, as case reports, or showing a different scenario with *A. baumannii* as the most common species [23–27].

Present results show worrisome levels of antibiotic resistance amongst *bla*_OXA−51_-positive isolates, with levels of nonsusceptibility higher than 78% in all cases, except ampicillin plus sulbactam and colistin. This issue agrees with the scenario described in Peru for *Acinetobacter* spp. and other clinically relevant pathogens [14]. Regarding colistin, 52 out of 53 isolates, irrespective of the presence or absence of *bla*_OXA−51_ were from HAI; this finding strongly suggests a direct pressure in hospital settings. An identical issue may be observed for the remaining antibacterial agents. Different reports have shown that the antimicrobial resistance levels of *A. baumannii* are higher than those of other members of the ACB complex [21, 28, 29]. In the absence of exact species identification, this finding agrees with the presence of significant differences in the resistance levels of *bla*_OXA−51_-positive and *bla*_OXA−51_-negative among our isolates. This, together with the chromosomal encoding of *bla*_OXA−51_, supports the common circulation of NBA in Peru. In this sense, the present data suggest that rapid detection of *bla*_OXA−51_ will contribute to an early initiation of empiric treatment until an exact antibiogram is obtained, leading to better patient management as well as more adequate use of antibacterial agents. Those isolates, species-specific, identified showed a full concordance between *bla*_OXA−51_ presence/absence and 16S rRNA or *rpoB* sequencing results. In agreement with the present results, previous reports have shown inaccuracies in the MALDI-TOF identification of *A. baumannii* [30]. In our study, additional issues such as low identity levels in several cases or the lack or low number of profiles of *Acinetobacter* spp. isolates circulating in Peru included in the MALDI-TOF database might also contribute to explaining this finding.

In our study, the *bla*_OXA−51_-positive isolates showed worrisome levels of antimicrobial resistance, warning about the need for both more cautious use of antibacterial agents to reduce the pressure of antimicrobial agents on bacteria, as well as the need for new therapeutic options, whether they be antibiotics or not.

The evaluation of the origin of the *bla*_OXA−51_-negative isolates showed that they were more frequently from outside metropolitan Lima and were from the period 2023 to 2024. While possible sample biases related to specificities of the health centers participating in the study, geographic imbalance, and the fact that samples were not collected within the same time frame in all centers cannot be ruled out, these results might be associated with differences in patient management or local antibiotic pressure on bacteria. In this sense, the period of the COVID-19 pandemic has been characterized by high use of antimicrobial agents in both hospital settings and the community [31, 32]. The end of the COVID-19 pandemic led to decreasing pressure on the healthcare system and, in parallel, a reduction in antibiotic use in hospital and community settings, thereby allowing the appearance (or re-appearance) of microorganisms with lower levels of antibiotic resistance in hospital environments. The low number of prepandemic isolates impairs a more in-depth analysis and verification of this issue.

Of note, the present results also showed that community-acquired infections related to both *bla*_OXA−51_-negative and -positive *Acinetobacter* showed lower levels of resistance than those from HAIs. This finding agrees with results previously published [24–26], probably being related to the antibiotic pressure exerted on hospital environments. Furthermore, while potential confounding variables such as prior hospitalization or treatment history may blur the HAI/community distinction, this result supports differences in the origin of isolates.

The main limitations of the present study are the nonparallel obtainment of isolates from different regions, hindering the interpretation of the evolution of *bla*_OXA−51_-negative isolates together with the low number of *bla*_OXA−51_-negative isolates and the disparity of the number of isolates among the different sources (metropolitan Lima/other regions). However, our data show that 24.6% of the more recent ACB complex isolates (period 2023–2024) were *bla*_OXA−51_-negative, highlighting the relevance of these pathogens in Peru. In addition, the possible acquisition of *bla*_OXA−51_ in a few isolates of NBA as well as the possible presence of *bla*_OXA−51_ defective *A. baumannii* cannot be ruled out [12, 13]. Nevertheless, the significant differences in antimicrobial resistance patterns, as well as in the community or HAI origin of *bla*_OXA−51_-negative and -positive *Acinetobacter*, clearly support the presence of true genetic differences between *bla*_OXA−51_ producers and nonproducers, with the most likely scenario being that these differences mirror the presence of different *Acinetobacter* species. In a few cases, the identification of *bla*_OXA−51_-negative isolates by automatized/biochemical techniques might be erroneous, and the isolates did not, in fact, belong to the *Acinetobacter* genus [33]. However, it should be taken into account that these are the standard technologies used to identify these microorganisms in a long series of health centers worldwide. Of note, in several cases, no data about biological samples, clinical outcome, or HAI/community origin were available, with this probably being related to the high pressure exerted over health centers during the COVID-19 pandemic. Similarly, no information about previous hospitalizations was recorded.

## 5. Conclusion

In summary, the amplification of *bla*_OXA−51_ in the isolates analyzed suggests that NBA is present throughout Peru. Despite limitations, the use of this approach may be useful to achieve early and more adequate therapeutic management of patients in those low- and middle-income areas where exact identification of *Acinetobacter* species is not possible. Further studies should focus on the unequivocal identification of species circulating in Peru.

## Figures and Tables

**Table 1:** Isolates included in the study.

Hospital	City	Area	Ubication	*N*	*bla*_OXA−51_		Year
					Present	Absent	
A	Lima	Metropolitan Lima	Coast	84	77	7	2018–2024
B	Lima	Metropolitan Lima	Coast	57	52	5	2023–2024
C	Lima	Metropolitan Lima	Coast	52	49	3	2021
D	Lima	Metropolitan Lima	Coast	24	16	8	2023–2024
E	Lima	Metropolitan Lima	Coast	17	16	1	2021–2024
F	Lima	Metropolitan Lima	Coast	12	10	2	2021–2023
G	Lima	Metropolitan Lima	Coast	11	8	3	2023–2024
H	Lima	Metropolitan Lima	Coast	6	5	1	2021–2023
nd^a^	Lima	Metropolitan Lima	Coast	2	1	1	2023–2024
I	Lima	Metropolitan Lima	Coast	1	1	0	2023
J	Callao	Metropolitan Lima	Coast	1	1	0	2023
K	Piura	Northern Peru	Coast	2	2	0	2021
L	Huánuco	Central Peru	Mountain	6	4	2	2023–2024
M	Iquitos	Northern Peru	Jungle	9	6	3	2020–2024
N	Tacna	Southern Peru	Coast	14	12	2	2023–2024

		Metropolitan Lima		267	236	31	*p*=0.0912
		Remaining Peru		31	24	7	
		Overall		298	260	38	

*Note: N*, number of isolates.

Abbreviation: nd, no data.

^a^These isolates were from other hospitals in the area and were referred to a participant center in Lima for bacterial identification.

**Table 2:** Temporal distribution of the isolates analyzed.

Period	*N*	*bla*_OXA−51_				*p*
		Present		Absent		
		ML	Other	ML	Other	
2018–2019	12	12	0	0	0	0.0003
2020–2021	122	111	5	6	0	
2022–2024	164	113	19	25	7	

*Note:* Other, remaining regions. Due to the low number of isolates, pre-COVID-19 pandemic isolates (2018–2019) were not considered in the statistical analysis.

Abbreviation: ML, metropolitan Lima.

**Table 3:** Comparison of different methods (MALDI-TOF, *rpoB* sequencing, 16S-rRNA sequencing, and *bla*_OXA−51_ presence) used to identify a subset of 30 isolates.

Comparison	*N*	MALDI-TOF	*rpoB*	16S	*bla*_OXA−51_
Concordance	14	*A. baumannii*	*A. baumannii*	*A. baumannii*	+
	1	*A. nosocomialis*	*A. nosocomialis*	*A. nosocomialis*	−
	1	*A. soli*	*A. soli*	*A. soli*	−
	1	*A. pittii*	*A. pittii*	*A. pittii*	−
	1	*A. lactucae*	*A. lactucae*	*A. lactucae*	−

Discordance	1	*A. lactucae*	*A. pittii*	*A. pittii*	−
	2	*A. baumannii*	*A. pittii*	*A. pittii*	−
	1	*A. pittii*	*A. baumannii*	*A. baumannii*	+
	1	*A. soli*	*A. pittii*	*A. pittii*	−
	3	*A. soli*	*A. baumannii*	*A. baumannii*	+
	2	*A. lactucae*	*A. baumannii*	*A. baumannii*	+
	1	*A. nosocomialis*	*A. pittii*	*A. pittii*	−

**Table 4:** Source of infection of recovered isolates as community and hospital origin.

Source	Community (25)							HAI (271)							*p*
	Neg (13)		Pos (12)		*p*	Overall		Neg (25)		Pos (246)		*p*	Overall		< 0.0001
	*N*	%	*N*	%		*N*	%	*N*	%	*N*	%		*N*	%	
Blood	2	15.4	0	0.0	NS	2	8.0	3	12.0	56	22.8	NS	59	21.8	NS
Respiratory	3	23.1	4	33.3	NS	7	28.0	15	62.5	127	51.2	NS	142	52.2	0.0224
Wound	0	0.0	0	0.0	—	0	0.0	2	8.3	10	4.0	NS	12	4.4	NS
Urine	8	61.5	6	50.0	NS	14	56.0	3	11.5	15	6.1	NS	18	6.6	< 0.0001
Catheter tip	0	0.0	0	0.0	—	0	0.0	0	0.0	8	3.2	NS	8	2.9	NS
Feces	0	0.0	0	0.0	—	0	0.0	0	0.0	4	1.6	NS	4	1.5	NS
Biological fluids^a^	0	0.0	0	0.0	—	0	0.0	2	8.3	11	4.4	NS	13	4.8	NS
Other	0	0.0	2^b^	16.7	< 0.0001	2	8.0	0	0.0	0	0.0	—	0	0.0	0.0068
No data	0	0.0	0	0.0	—	0	0.0	0	0.0	14	5.6	—	14	5.1	NS

*Note: N*, number of isolates; Neg, *bla*_OXA−51_-negative isolates; Pos, *bla*_OXA−51_-positive isolates; —, not determined. In two cases (both *bla*_OXA−51_-positive), no information about the community or HAI origin was recorded.

Abbreviations: HAI, hospital-acquired infection; NS, nonsignificant.

^a^Cerebrospinal, biliary, peritoneal, and undetermined fluids.

^b^Two maternal milk samples.

**Table 5:** Antibiotic nonsusceptibility levels.

A.A.	*bla*_OXA−51_-negative (*n* = 38)				*bla*_OXA−51_-positive (*n* = 260)				*p*
	I	R	NS	%	I	R	NS	%	
AMS	3	5	8	20.5	86	65	151	58.1	< 0.0001
PTZ	5	11	16	42.1	8	214	222	85.4	< 0.0001
CTX	20	14	34	89.5	26	222	248	95.4	0.1319
CAZ	1	13	14	36.8	3	217	220	84.6	< 0.0001
FEP	4	10	14	36.8	60	153	213	81.9	< 0.0001
IMP	1	14	15	39.5	0	224	224	86.2	< 0.0001
MEM	3	15	18	47.4	0	224	224	86.2	< 0.0001
CIP	2	7	9	23.7	0	224	224	86.2	< 0.0001
LVX	1	7	8	21.1	2	217	219	83.6	< 0.0001
GM	3	7	10	26.3	13	201	214	82.3	< 0.0001
AMK	1	5	6	15.8	13	192	205	78.8	< 0.0001
COL	—	6	6	15.8	—	47	47	18.1	0.8242

*Note:* I, intermediate; R, resistant; %, percentage of nonsusceptibility; AMS, ampicillin plus sulbactam; PTZ, piperacillin plus tazobactam; CTX, cefotaxime; CAZ, ceftazidime; FEP, cefepime; IMP, imipenem; MEM, meropenem; CIP, ciprofloxacin; LVX, levofloxacin; GM, gentamicin; AMK, amikacin; COL, colistin.

Abbreviations: A.A, antimicrobial agent; NS, nonsusceptible.

**Table 6:** Distribution of *bla*_OXA−51_-negative and *bla*_OXA−51_-positive *Acinetobacter* spp. among community and hospital-acquired infections.

	*bla*_OXA−51_-negative (*n* = 38)								*p*	*bla*_OXA−51_-positive (*n* = 258)								*p*
	Comm (*N* = 13)				HAI (*N* = 25)					Comm (*N* = 12)				HAI (*N* = 246)				
	I	R	NS	%	I	R	NS	%		I	R	NS	%	I	R	NS	%	
AMS	0	2	2	15.4	3	3	6	24.0	—	1	3	4	33.3	85	62	147	59.8	—
PTZ	0	2	2	15.4	5	9	14	56.0	0.0356	0	5	5	41.7	8	209	217	88.2	0.0003
CTX	7	4	11	84.6	13	10	23	92,0	—	2	6	8	66.7	22	216	238	96.7	0.0011
CAZ	0	2	2	15.4	1	11	12	48.0	—	0	5	5	41.7	3	212	215	87.4	0.0004
FEP	1	3	4	30.8	3	7	10	40.0	—	0	5	5	41.7	60	148	208	84.6	0.0013
IMP	0	2	2	15.4	1	12	13	52.0	0.0394	0	7	7	58.3	0	217	217	88.2	0.0119
MEM	0	2	2	15.4	1	12	13	52.0	0.0394	0	7	7	58.3	0	217	217	88.2	0.0119
CIP	1	1	2	15.4	1	6	7	28.0	—	0	6	6	50.0	0	218	218	88.6	0.0018
LVX	0	2	2	15.4	1	5	6	24.0	—	0	6	6	50.0	2	211	213	86.6	0.0038
GM	1	3	4	30.8	2	4	6	24.0	—	0	7	7	58.3	13	194	207	84.1	0.0360
AMK	0	1	1	7.7	1	4	6	24.0	—	0	6	6	50.0	13	186	1999	80.9	0.0194
COL	—	0	0	0.0	—	6	6	24.0	—	—	1	1	8.3	—	46	46	18.7	—

*Note:* Comm, community; I, Intermediate; R, resistant; %, percentage of nonsusceptibility; AMS, ampicillin plus sulbactam; PTZ, piperacillin plus tazobactam; CTX, cefotaxime; CAZ, ceftazidime; FEP, cefepime; IMP, imipenem; MEM, meropenem; CIP, ciprofloxacin; LVX, levofloxacin; GM, gentamicin; AMK, amikacin; —, nonsignificant. In two cases, no information about the community or HAI origin was recorded. These isolates were *bla*_OXA−51_-positive and susceptible to all tested agents, except CTX (intermediate in both cases).

Abbreviations: HAI, hospital-acquired infection; NS, nonsusceptible.

## Data Availability

The data that support the findings of this study are available from the corresponding author upon reasonable request.
